# Should tourists care more about invasive species? International and domestic visitors’ perceptions of invasive plants and their control in New Zealand

**DOI:** 10.1007/s10530-022-02890-8

**Published:** 2022-09-13

**Authors:** Brent Lovelock, Yun Ji, Anna Carr, Clara-Jane Blye

**Affiliations:** 1 Department of Tourism, Centre for Recreation Research, University of Otago, Dunedin, New Zealand; 2 Faculty of Kinesiology, Sport, and Recreation, University of Alberta, Edmonton, Canada

**Keywords:** Invasive alien species, Invasive plants, Tourists, Wild conifers, Russell lupins, New Zealand

## Abstract

Tourism has been implicated in the spread of invasive species, not only through physical means but through invasive species being perpetuated in destinations as part of the tourism landscape. This study reports on a survey of 238 domestic and international tourists visiting the south of New Zealand, with a focus on their knowledge of and attitudes to the management of two invasive plants: wild conifers and Russell lupins. Both plants have profound ecological, economic and environmental impacts but are also increasingly a part of the tourist landscapes in the study region. The survey found significant differences between domestic and international visitors in their levels of ecological knowledge about the invasive plants, with domestic visitors having greater awareness. However, there were also significant differences between international visitors according to origin and ethnicity, with Asian visitors showing lower awareness and also lower willingness to support eradication of the invasives, even after being provided information on the ecological impact of the species. Participants also responded differently to the two species, being less willing to support eradication of the attractive Russell lupin, compared to wild conifers. There are implications for management in terms of the messaging that may be required for different visitor groups around invasive species control. The study also points to the challenge of developing support for the management of charismatic plant species such as Russell lupin that are now firmly located within the tourism domain.

## Introduction

To date, much social dimensions of IAS (Invasive Alien Species) research has focused on the general public and local communities of interest, with less emphasis placed on the perceptions and understandings of visitors to a site of invasion (Nikodinoska et al 2014). But given that IAS management often draws upon broader social and political support than can be provided by the residents of the immediate area effected by the invasion, it is pertinent to expand the boundaries of who we consider to be IAS stakeholders, and to explore the level of IAS awareness of ‘outsiders’. This is particularly the case if we think of these individuals as external stakeholders, whose support may be desirable or essential to IAS management.

A case in point is the control of invasive conifers in New Zealand, an estimated $166 M programme (Wyatt 2018) over tracts of land which many or most New Zealanders have never visited. Much of this IAS problem in New Zealand is regionally concentrated in the lower South Island, hundreds of kilometres away from the main population centres of the nation. While many New Zealanders would only be vaguely aware of these southern landscapes, generally as tourism destinations, all tax-paying New Zealanders are being called upon to contribute to the costs of invasive conifer control. Of course many may be happy to do so, bearing in mind that while they may be regionally disconnected from the IAS problem, a sense of ‘ecological patriotism’ (Warren 2012) may apply and manifest in terms of support for control measures. A second group of ‘outsiders’, however, perhaps even further disconnected, at least in distance, comprises the roughly four million international visitors that arrive each year (prior to Covid-19) – roughly the same number as the resident population of New Zealand. While most of these arrive from tourism generating regions thousands of kilometres distant, an irony is that this group of international visitors may have greater contact with these landscapes, and the IAS within them, than many New Zealanders. This comes about through their exposure to pre-travel touristic promotional imagery (formal and informal) and then through their actual travel along the touristic itineraries where these IAS may be encountered.

While international tourists are not being asked to contribute financially to IAS control, they are IAS stakeholders as some IAS may form important components within tourists’ itineraries, contributing to their experiences and overall satisfaction. In New Zealand, for example, some invasive plant species feature strongly within official tourism promotional material, and images of these species are also disseminated and perpetuated through tourist-related social media. In this sense, tourists, both domestic and international, have a pecuniary interest in some IAS.

Yet in New Zealand, and internationally, with a few notable exceptions (e.g. Ansong and Pickering 2015; Bravo-Vargas et al. 2018; Lovelock, 2007; Nanayakkara et al. 2018; Sharp et al. 2012) we have not fully involved tourists as participants within our research on the social dimensions of IAS, as the focus has been, understandably, upon local residents’ and immediate resource users’ relationships with IAS. A consequence of this is that we know little about tourists’ IAS awareness and knowledge, or their attitudes with respect to IAS management. Nor do we know a lot about how visitors’ perceptions may be linked to the particular characteristics of individual invasive species. Tourism, as New Zealand’s major export earner (until the impacts of Covid-19) relies heavily on its endemic fauna and flora, its landscape and the nation’s 100% pure and green image (Beattie 2011; Hayes and Lovelock 2017). Tourism is often negatively affected by biological invasions and also significantly contributes to such invasions (Hall et al. 2011; Anderson et al. 2015), pointing to the need for tourists’ (and the tourism industry’s) relationships with IAS to be taken into consideration.

## The role of place of residence

Shackleton and colleagues (2019) propose a conceptual framework identifying six broad-scale core factors with a wide range of underlying factors that can influence perceptions of invasive alien species (IAS) and their management. Among these are the characteristics of the individuals themselves and the attributes of the invasive alien species itself. Since perceptions are socially and culturally constructed, individuals with different demographic profiles are expected to have different perceptions of IAS and attitudes toward their management (Shackleton et al. 2019). One such demographic variable is place of residence although this has yet to be fully explored in IAS social dimensions studies: i.e. where the individual lives, and connected with this, their social and cultural background.

It is reasonable to assume that the level of knowledge about particular IAS will be associated with place of residence, as exposure to IAS and associated environmental messaging will likely be greater for those residing within the ‘invasion zone’ of that IAS (i.e. the same region, state or country) compared to those from further afield (e.g. overseas) who may not have had this exposure. Likewise, by association, support for IAS control measures will also vary by place of residence. In support of this assumption Lovelock (2007) found variation between domestic tourists and international tourists in terms of their level of ecological knowledge and attitudes towards IAS and their management in New Zealand. Similarly, Zhang and colleagues (2021) in their qualitative study of international visitors in eco-sanctuaries in New Zealand encountered examples of where nationality appeared to influence visitors’ knowledge of, and attitudes towards, particular IAS. However, neither of the above studies was aimed at segregating international visitors by nationality or other means, Lovelock (2007), for example, treated international visitors as a single cohort. Further studies have identified place of residence as a factor in perceptions of IAS, for example rural vs urban residence was related to visitors’ understanding of aquatic invasive species in Canada (Nanayakkara et al. 2018), that study, however only addressed domestic visitors. Pissolito et al. (2020) found differences between visitors of local, regional and national origin in regard to their perceptions of pine-invasion in Argentinian Patagonia, and likewise in their level of support for management. Similarly, Junge et al. (2019) found regional differences in willingness to pay for invasive plant interventions between the German, French and Italian-speaking parts of Switzerland, associating these regional differences with a higher problem awareness among some groups.

## Knowledge of IAS

Previous studies have shown that environmental strategies and conservation programmes which aim to raise public awareness are vital to IAS management (Shackleton et al. 2007). Such awareness can also contribute more directly to IAS management through leading to more conservation-friendly behaviours with respect to spreading and reporting of IAS (Caffrey et al. 2014; Novoa et al. 2017; Cole et al. 2019).

However, studies have shown that the public’s awareness of IAS is low (Sharp et al. 2017). Sharp et al. (2012) found that most visitors to a natural site were only slightly familiar with IAS and even less aware of their impact. Other studies suggest that IAS are not a concern to the public unless they pose a threat to nature, the economy of an area, or human health (Verbrugge et al. 2013), and that support is only shown for removal of those invasive plants that provoke serious problems and costs (Lindemann-Matthies 2016). This preference for the eradication of only economically damaging species is also evident in other studies (Bardsley and Edward-Jones 2006; Bremner and Park 2007; García-Llorente et al. 2008). Further to this, there is evidence that the native/introduced distinction may not be important to the general public- with perceived ecological and economic threat, rather than non-nativeness per se, found to influence attitudes towards species management (van der Wal et al. 2015).

What is known, however, is that enhanced knowledge about the effects of IAS can influence attitudes towards their acceptability and regarding their management. An empirical study of the public’s concerns about invasive pines in New Zealand (Gawith et al. 2020) found that large numbers of survey respondents initially reported that they held no opinion about the incursion of wild conifers on sites of significance in their areas. However, participants’ concerns about invasive pines increased when they were presented with scientifically credible information about likely rates of spread (see also Bravo-Vargas et al. 2019). Similarly, Novoa et al. (2017) found that providing knowledge to the public about the harmful effects of IAS increased support for management, even after receiving only a “limited amount of information provided on the origin and negative impacts of the target species” (p 3701).

## Aesthetics of the invasive

Notwithstanding the above findings, there is some evidence that the attributes of a species, such as how long they have been present in the landscape may hamper public acceptance of IAS control. In many places, IAS have existed for a long time, gaining public acceptance as part of the local landscape, culture, and identity, and contributing to the area’s ‘sense of place’ (Shackleton et al. 2019). Such IAS may have been perceived as native species by locals, tourists and recreationists (Fisher and Van Der Wal 2007; Hall and Baird 2013). Some invasive plant species have become symbols of destinations e.g. *Pinus* in Twizel the ‘Tree Town’, New Zealand, Jacaranda (*Jacaranda mimosifolia*) in Pretoria, South Africa (Dickie et al. 2014), and Albizzia *(Falcataria moluccana)* in Hawaii, USA (Niemiec et al. 2017).

A further complicating factor is that the public might not support the removal of ‘beautiful’ invasive plants, i.e., plants with ornamental value (Veitch and Clout 2001). Lindemann-Matthies (2016) showed that with the increasing appeal of an invasive plant, agreement for its removal decreased, the public being unwilling to remove plants that were established ornamentals; “Overall, willingness to remove an [invasive plant species] and to report it to the authorities decreased with increasing desirability (and thus beauty) of a species” (2016, p 15). Lindemann-Matthies (2016) found that women were generally more in favour than men of visually appealing plants but that ‘showy’ plants were favoured by all their study participants. However, Junge and colleagues (2019) found that after providing information on the invasiveness and ecological impact of a set of invasive plants (which included some attractive flowering plants), respondents’ aesthetic preferences for all species decreased significantly and that they also showed stronger support for more intensive control of the plants.

## The invasive plants in this study

Two invasive plants were the focus of this study, wild conifers and Russell lupins, the latter having particularly high aesthetic value, but both commonly encountered in the touristic landscapes of the study region. Wild conifers (also known as wilding conifers or wilding pines) have become one of New Zealand’s most important and costly environmental weeds over the last hundred years (Gawith et al. 2020). These conifers (a mix of North American and European species within the Pinacae family, including *Pinus contorta* (Lodgepole pine), *Pinus nigra* (Corsican pine) *Pinus radiata* (Radiata pine), *Pseudotsuga menziesii* (Douglas Fir) and *Larix decidua* (European larch), have spread through seeding and are currently present across 1.7—1.8 million hectares in New Zealand (Nunez et al. 2017). They are predicted to expand in area at a rate of 5% per year (Greenaway et al. 2015), out-competing native plant life and leading to substantial ecosystem change (Kirk 2017; Dechoum et al. 2019). They also invade pastoral farmland, affect water regimes, increase fire risk, and adversely affect landscape values and visual amenity (Froude 2011) (New Zealand Wilding Conifer Management Group 2014; Dechoum et al. 2019). Some benefits of wildling conifers have been noted, for example, their contribution to increasing carbon storage for greenhouse gas mitigation and reducing dryland erosion (Mason et al. 2017; Nunez et al. 2021). Conifers have spread through high altitude native grassland in both study areas which are focal points for the current national programme of wild conifer control.

Russell lupin (*Lupinus* × *russellii)* is a decorative perennial garden plant that is a hybrid of a species native to North America (where lupins are also considered a pest in some locations (National Park Service 2021)). This plant is present in both study areas and is a significant problem in Te Manahuna Mackenzie Basin where it rapidly invades shingly braided river systems that are characteristic of this area. There it modifies river flows, reduces nesting site availability for a number of endangered birds, and provides cover for invasive predators (cats, mustelids, hedgehogs) (Caruso et al. 2013). Large amounts of seed are spread by water, and also by humans purposefully distributing them along roadsides Lupin’s current wide range across Te Manahuna originates from the 1930s when initially planted in the gardens of high country farms. From that time, seeds were then deliberately spread along the roads of the area to ‘beautify’ the landscape (Scott 1989; InspiredNZ.com 2021; Weedbusters 2021). Local legend has it that seeds were also given by bus drivers to their tourist passengers to distribute during their stays in the region. Swathes of land covered in Russell lupins in bloom now feature prominently in the official tourism imagery and among visitors’ social media imagery (Authors in preparation) and seeds continue to be sold in nurseries and tourist shops. Lupins are valued not only by tourists, but also by some famers in the area as a nitrogen fixing plant and source of sheep fodder in difficult dryland soils (Scott 1989).

The study had two main aims, the first was to extend the social dimensions of invasive species research into a relatively neglected set of participants, tourists, and to investigate the role of place of residence by comparing domestic and international visitors’ perceptions of invasive plants. The second aim was to investigate whether knowledge of the ecological impacts of invasive plant species would change tourists’ views towards the plants and their control, especially if the plant had high aesthetic value.

## Methods

A questionnaire was developed for the purpose of the study, with some items broadly developed from Lovelock (2007) but more generally informed by the social dimensions literature on IAPS (e.g. Lindemann-Matthies 2016; Bravo-Vargas et al. 2019). This paper reports on one component of a larger survey which explored knowledge of and preferences for a range of plant species in New Zealand, along with attitudes toward IAPS and their control (Authors in preparation).

The first section of the questionnaire aimed to determine participants’ awareness of ecological problems caused by IAS and participants’ perceptions of IAS through focusing on two contentious plants: wild conifers and Russell lupins. In this section, visitors were presented with a photo of a landscape with the selected plant and were asked how they felt about the plant in the landscape (Do you think that this plant makes this landscape more or less attractive?), and whether they were aware of any ecological problems associated with the plant (Are you aware of any ecological problems that this plant may cause?). An explanation of the ecological impacts caused by these plants was then provided for participants (Wild Conifers: If we were to tell you that this introduced plant invades native tussock grasslands and causes problems for the diversity of native animals and plants would this change your opinion about the plant, and if so, how? Rusell lupins: If we were to tell you that this plant smothers natural rocky river beds and causes problems for native birds that usually nest in river beds would this change your opinion about the plant, and if so, how?). Participants were then asked whether they would change their opinions about the plants and support the control of these IAS by selecting from four possible responses: (1) Yes, I would support its eradication; (2) Yes, but I still like to see it in the landscape; (3) No, I think it looks lovely; (4) No, I think that we should just accept it as part of the NZ ecosystem. The second section of the questionnaire contained questions related to personal and socio-demographic information, including place of origin, age, gender and ethnicity.

Considering that visitors from China comprise the second largest inbound market for New Zealand and the single biggest non-English speaking market (MBIE 2018), it was decided that the questionnaire would be conducted in both English and Mandarin. The questionnaire was designed in English and then translated into Mandarin, with independent back-translation undertaken to check consistency of terms used. A pilot study was run with the Mandarin version of the questionnaire to ensure Chinese respondents could adapt to the scale and constructs of the survey. Cultural and linguistic diversity challenges cross-cultural research, with individuals from different cultures often having different interpretations and experiences with the scales and constructs (McGorry 2000). Twelve Chinese individuals were invited to pretest the survey. Interviews were then conducted with each participant to understand whether individuals had any difficulties in understanding the survey contents. Feedback was encouraged and collected for survey improvement, however no key issues or problems were indicated by respondents.

As this study required collection of primary data from human participants, ethical approval was gained prior to the distribution of the pilot and main surveys. The broad aim of the research project was disclosed to participants through an Information Sheet provided on site. Participants were assured anonymity in the final report and could choose, at any time, to withdraw from the survey.

**Fig. 1:**
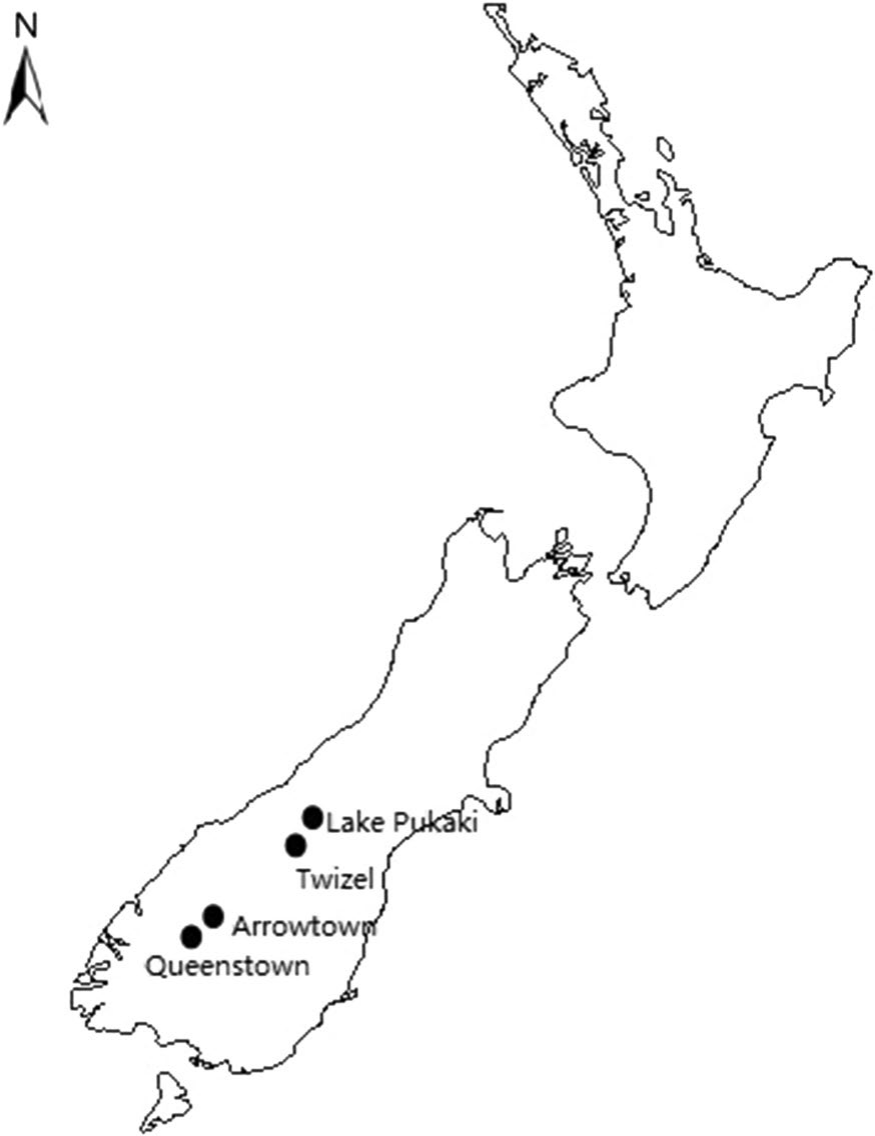
Survey Locations

The distribution of the survey was carried out during the summers of 2019 and 2020 at popular viewpoint sites near Queenstown and Arrowtown in the Queenstown Lakes district, and near Twizel and Lake Pukaki in Te Manahuna Mackenzie Basin (Fig. 1) (and see Study Setting below). A convenience sampling technique was used to recruit the next available visitor who was willing to take part in the survey (Etikan et al 2016). Surveys were self-completed mainly via iPad (whilst hard copies were available for respondents who preferred this medium). Two researchers on site (one Mandarin speaking) distributed the survey to visitors. Fruit and candy were available as incentives for survey participants.

SPSS (Statistics Package for the Social Sciences version 24) was used for data analysis. Descriptive techniques were utilized to compute frequencies, means for responses within each response category for all questions by socio-demographic groups. Differences between groups by socio-demographics (place of residence; nationality, ethnicity; gender; age) were examined utilizing comparative analysis (Chi-Square test) all assumptions being satisfied for this test (McHugh 2013).

The study took place within two touristic settings in the South Island of New Zealand. The first of these, Te Manahuna, the Mackenzie Basin, is located in the central high-country area of the South Island, comprising an inter-montane basin, and large areas of mountainous terrain. The second site, the Queenstown Lakes district is in the mountainous interior region of the Otago region. Both areas support a unique high-country landscape with high aesthetic and historical and cultural values, making them very popular for a variety of tourist activities (Gawith et al. 2020). Both areas also have cultural significance to the indigenous Māori of Te Rūnanga o Ngai Tāhu (the local tribal group (iwi)). The iwi has strong traditional associations to Whakatipu-waimaori (Lake Wakatipu) in the Queenstown Lakes and to Aoraki/Mt Cook in Te Manahuna, which is the highest mountain of New Zealand (Greenaway et al. 2015). In 2018, Aoraki/Mt Cook was reported to be visited by more than one million visitors with Queenstown receiving 3.9 million visitors in 2019 (Sage 2019).

In both study areas the flora is significantly modified by fire and farming, but large areas of high altitude native grassland remain. In this way, the study areas are reflective of many New Zealand landscapes which now comprise mixtures of native species and invasive alien plants (De Lange et al. 2009). New Zealand as a whole has been profoundly affected by invasions of exotic species, particularly since the early nineteenth century, following European colonisation (Beattie 2011).

## Results

Socio-demographic profile

Of the 238 surveys collected, seven were either not fully completed or there was incomplete demographic information, reducing the effective sample size to 231. The majority (about three-quarters of the sample) were international visitors, with New Zealand domestic visitors comprising the remaining one-quarter of the sample (Table 1). The sample, in terms of domestic and international visitor composition was broadly reflective of pre-Covid-19 visitation to this region of New Zealand (MBIE 2018). There were slightly more female respondents than male respondents. The number of ‘Other’ gender group respondents (n = 3, 1.3%) was limited in the sample, precluding this as a category for comparative statistical analysis. Visitors in the 18–29 years range formed the largest age group comprising over one-third of the sample, followed by the 30–39 years old group. Due to lower numbers of participants in the two oldest groups (60–69years and 70 +) these were combined to create a larger group for the purpose of statistical analysis.

**Table 1:** Survey participant profile

	n	Percent (%)
Visitor Status		
NZ Visitors (Domestic)	56	24.2
International Visitors	175	75.8
Nationality^a^		
Australia	24	12.8
UK or Europe	56	29.9
USA or Canada	23	12.3
China	45	24.1
Other Asia	26	13.9
Other	13	7.0
Ethnicity		
European	111	48.1
Asian	98	42.4
Other	22	9.5
Age		
18–29	82	35.7
30–39	62	27.0
40–49	34	14.8
50–59	28	12.2
60 +	24	10.4
Gender		
Male	104	45.2
Female	123	53.5
Other	3	1.3
Total	231	100.0

^a^12 respondents recorded dual citizenship between New Zealand and another country

Of the international visitors, the majority came from the UK or Europe, followed by China, then Other Asia, Australia, and USA or Canada. Visitors were mainly of two ethnicities with Europeans comprising just under half of the sample, followed by Asians at 42.4%. There were limited numbers of Māori and Pasifika participants in this study (about 2%), precluding comparative statistical analysis of these groups, thus it was decided to combine them with the ‘Other’ ethnicity group.

### Visitors’ attitudes toward wild conifers

An image of a natural high country landscape typical of the study region, but with wild conifers present was shown to participants. A considerable number of participants (n = 96, 41.9%) said that wild conifers made the landscape more attractive (Table 2). Only one-third of the participants were aware of the ecological problems caused by wild conifers (n = 75, 33%). New Zealand visitors had greater awareness of the ecological problems caused by wild conifers (n = 31, 55.4%) than did international visitors (n = 42, 25%) (χ^2^ = 17.62, df = 1, p < 0.001). However, about one third of New Zealand visitors (n = 19, 33.9%) said the wild conifers made the landscape more attractive.

**Table 2:** Wild conifers: Visitors’ initial perceptions and knowledge of, and subsequent attitudes towards, following the provision of invasiveness information

	n	Pre-information		Post-information			
		Attitude	Knowledge	Opinion (%)			
		Find attractive (%)	Awareness of ecological problem (%)	a	b	c	d
Visitor Status							
All Visitors	231	41.9	33.0	72.7	17.6	3.5	6.2
NZ Visitors	56	33.9	55.4	73.2	17.9	5.4	3.6
International Visitors	175	44.7	25.0	72.0	17.9	3.0	7.1
Place of Origin							
Australia	24	37.5	47.0	83.3	12.5	4.2	0.0
UK or Europe	56	32.7	35.7	80.4	14.3	1.8	3.6
USA or Canada	23	40.9	26.1	90.9	4.5	0.0	4.5
China	45	55.6	11.6	59.5	19.0	4.8	16.7
Other Asia	26	61.5	12.0	42.3	42.3	7.7	7.7
Other	13	38.5	46.2	69.2	7.7	15.4	7.7
Ethnicity							
European	111	31.2	43.6	83.8	12.6	0.9	2.7
Asian	98	54.1	17.9	60.6	22.3	5.3	11.7
Other	22	40.9	54.5	68.2	22.7	9.1	0.0
Age							
18–29	82	39.0	30.9	75.6	18.3	1.2	4.9
30–39	62	53.2	28.3	68.3	16.7	6.7	8.3
40–49	34	38.2	29.4	57.6	27.3	6.1	9.1
50–59	28	40.7	46.4	85.7	14.3	0.0	0.0
60+	24	30.4	39.1	82.6	8.7	0.0	8.7
Gender							
Male	104	36.9	42.2	74.8	14.6	4.9	5.8
Female	123	45.9	25.6	70.8	20.8	1.7	6.7

a Yes, I would support its eradication

b Yes, but I still like to see it in the landscape

c No, I think it looks lovely

d No, I think that we should just accept it as part of the NZ ecosystem

After participants were provided with the statement about the ecological impacts of wild conifers, over 90% of the participants expressed a changed opinion about wild conifers, the majority saying they would support the eradication of wild conifers (n = 165, 72.7%). About 18% of participants said that while they had changed their opinion regarding wild conifers, they would still like to see wild conifers in the landscape. A small proportion (less than 10%) of participants retained their initial views, responding that ‘*wild conifers look lovely’* (n = 8, 3.5%) or ‘*we should just accept [wild conifers] as part of the NZ landscape*’ (n = 14, 6.2%). This response pattern was similar across domestic and International visitors.

When international visitors were analysed according to place of residence (nationality), those from Asian countries demonstrated significantly lower awareness of the ecological problems associated with wild conifers, compared with participants from other countries (χ^2^ = 17.24, df = 5, p = 0.004). After the ecological impacts of wild conifers were explained, the majority of participants from each place of origin expressed a changed opinion towards wild conifers. However, the opinions varied between visitors from Asian countries and other countries (χ^2^ = 33.35, df = 15, p = 0.004). The majority of visitors from non-Asian countries (Australia, UK or Europe, USA or Canada) expressed a changed opinion towards wild conifers and most of them supported eradication. However, much fewer participants from Asian countries supported the eradication of wild conifers. There were more participants from Asian countries who said they would still like to see wild conifers in the landscapes than non-Asian countries. With those participants who retained their opinion about wild conifers, there was a much higher percentage of participants from Asian countries than visitors from other countries who thought wild conifers should be accepted as part of the New Zealand ecosystem.

When analysed by ethnicity, the results mirrored the findings by place of residence (nationality). Asian participants showed higher preferences for the landscape with wild conifers than did European participants (χ^2^ = 11.11, df = 2, p = 0.004) and had a lower awareness of ecological problems caused by wild conifers than did European participants (χ^2^ = 16.97, df = 2, p < 0.001). After the impacts of wild conifers were explained, the majority of participants across all ethnic groups expressed a changed opinion about wild conifers, but opinions varied across different ethnic groups, Asian participants responding differently to participants of other ethnic groups (χ^2^ = 20.06, df = 6, p = 0.003). There was a higher proportion of European participants (n = 93, 83.8%) who supported the eradication of wild conifers compared to Asian participants (n = 57, 60.6%). There was also a higher percent of Asian participants (n = 21, 22.3%) than European participants (n = 14, 12.6%) still preferring landscapes with wild conifers or who thought wild conifers should be accepted as part of the New Zealand ecosystem.

Participants’ preference for landscapes with wild conifers varied across different age groups. Among all age groups, participants in the 60 + years group showed the lowest acceptance of wild conifers. Participants in the 30–39 years old showed the highest acceptance of wild conifers. Participants in the 50–59 years old showed the highest awareness of ecological problems caused by wild conifers, and the 30–39 years old group the lowest awareness. However neither of these findings were statistically significant. After the impacts of wild conifers were explained to participants the responses suggest that older participants (50 + years) showed more support for eradication, but this was not statistically significant.

Female participants showed a higher preference for the landscape with wild conifers (n = 56, 45.90%) than males (n = 38, 36.89%). Female participants also showed a lower awareness of ecological problems (n = 31, 25.62%) caused by wild conifers than male participants (n = 43, 46.12%) (χ^2^ = 6.83, df = 2, p = 0.033) There were no significant differences in wild conifer preference, knowledge or post-information responses between gender groups.

### Visitors’ attitudes toward Russell lupin

When participants were presented the image of the landscape with Russell lupins they showed a high preference for this landscape (n = 220, 96.1%), this being evident across both domestic and international visitors. Participants also showed relatively low awareness (n = 44, 19.47%) of ecological problems caused by Russell lupins (Table 3), but with New Zealand domestic visitors showing higher awareness than international visitors (χ^2^ = 13.69, df = 2, p < 0.001).

**Table 3:** Russell lupins: Visitors’ initial perceptions and knowledge of, and subsequent attitudes towards, following the provision of invasiveness information

	n	Pre-information		Post-information			
		Attitude	Knowledge	Opinion (%)			
		Find attractive (%)	Awareness of ecological problem (%)	a	b	c	d
Visitor Status							
All Visitors	231	96.1	19.5	42.9	43.3	7.6	6.3
NZ Visitors	56	94.6	36.4	42.6	46.3	7.4	3.7
International Visitors	175	96.5	13.7	42.5	42.5	7.8	7.2
Place of Origin							
Australia	24	87.5	25.0	58.3	33.3	8.3	0.0
UK or Europe	56	98.2	17.9	51.8	41.1	5.4	1.8
USA or Canada	23	100.0	9.1	45.5	40.9	4.5	9.1
China	45	93.2	7.0	33.3	47.6	4.8	14.3
Other Asia	26	96.2	16.0	25.0	41.7	25.0	8.3
Other	13	100.0	30.8	30.8	38.5	15.4	15.4
Ethnicity							
European	111	96.4	24.5	54.5	40.0	2.7	2.7
Asian	98	94.8	14.9	32.6	35.7	10.9	10.9
Other	22	100.0	11.1	33.3	38.9	22.2	5.6
Age							
18–29	82	93.9	14.8	48.8	37.8	8.5	4.9
30–39	62	100.0	13.3	32.8	54.1	6.6	6.6
40–49	34	91.2	11.8	32.3	45.2	12.9	9.7
50–59	28	96.3	42.9	51.9	44.4	0.0	3.7
60+	24	93.9	31.8	54.5	31.8	4.5	9.1
Gender							
Male	104	94.2	22.5	47.1	36.5	7.7	8.7
Female	123	97.5	16.7	38.8	50.0	6.9	4.3

a Yes, I would support its eradication

b Yes, but I still like to see it in the landscape

c No, I think it looks lovely

d No, I think that we should just accept it as part of the NZ ecosystem

After the impacts of Russell lupins were explained, the majority of participants across different groups expressed a changed opinion about Russell lupin. However, in contrast with wild conifers, for those who changed their opinion, less than half supported eradication of Russell lupin (n = 96, 42.5%), and nearly half of the participants said they would still like to see Russell lupins in the landscape (n = 97, 42.5%). This attitude pattern was similar across both domestic and international visitor groups. There was a small proportion of participants (less than 10%) across both domestic and international visitor groups who did not support the eradication of Russell lupin for the reasons that “it looks lovely” or that “we should just accept it as part of the NZ ecosystem”. There were no significant differences regarding the post-information attitude patterns between the New Zealand visitor group and international visitor group.

Participants from a range of origins showed high preferences for the landscape with Russell lupins and generally low awareness of ecological problems caused by Russell lupins. For some international visitor groups (China, and USA or Canada) awareness of ecological problems was less than 10%. After the impacts of Russell lupins were explained to participants, the majority of participants across all groups expressed a changed opinion about Russell lupins. There were no significant differences in post-information attitude patterns by place of origin.

However, when analysed by ethnicity there were significant differences in post-information attitude patterns regarding Russell Lupins (χ^2^ = 21.041, df = 6, p = 0.002), with participants from the Asian and Other ethnicity groups having significantly different attitude patterns regarding Russell Lupins compared with visitors of European ethnicity. Over half of the European ethnicity participants supported eradication compared to one third of Asian and Other participants. A higher proportion of participants of the Asian and Other groups retained their positive opinions of Russell lupin, responding that it should be accepted as part of the ecosystem.

Russell lupins received high acceptance across all age groups, however, older participants (50 + years) had higher awareness of the ecological problems caused by Russell lupins than did younger participants (χ^2^ = 15.96, df = 4, p = 0.003) After the impacts of Russell lupins were explained, the majority of participants across different age groups expressed a changed opinion about Russell lupins, with no significant differences between age groups. Similarly, both males and female participants showed high preferences for the landscape with Russell lupins. There were no significant differences in preferences, knowledge or post-information attitude patterns between genders.

## Discussion

Although visitors often travel to seek experiences associated with a high level of naturalness or wildness in destinations, they are not necessarily aware of environmental problems such as invasive plant species that significantly undermine the integrity of the natural environment and biodiversity. Or, if they are aware, this may not necessarily translate into a high level of concern and support for management (Pissolito et al. 2020). In relation to the first aim of the paper, to extend the social dimensions of invasive species research to tourists, including international visitors, our results revealed that most visitors had a low awareness of ecological problems associated with our two focus invasive plants—wild conifers and Russell lupins. The data also reveals that New Zealand domestic visitors have higher levels of awareness than international visitors. However, surprisingly, only about half of our New Zealand domestic visitors were aware of the ecological problems caused by wild conifers (despite widespread campaigns and media publicity about conifer control in New Zealand), and only one-third were aware of the impacts of Russell lupins.

Along with this low level of awareness, was a relatively high level of acceptance overall for these species within our landscapes. This pattern (low-moderate awareness of the ecological problems of the species and moderate-high acceptance of the species in the landscape) was repeated across place of residence, ethnicity and other demographic groupings, however there were some significant differences. In general, international visitors from Asian countries were more accepting of the two invasive species in the landscape than were visitors from other countries. Similarly those of Asian ethnicity held significantly more positive attitudes towards both invasive species, and these attitudes were more resistant to change (after participants were provided information about the negative impacts of the species).

Previous studies have shown that attitudes to invasives and their control can vary according to visitor type (e.g. Pissolito et al. 2020 for local/regional/national visitors; Bravo-Vargas 2019 visit frequency; Lovelock 2007 for international/domestic), and also that there may be ethnic variations in preferences for the natural environment and landscapes (Buijs et al. 2009). This study found that visitors’ perceptions and attitudes toward invasive plants are related to both place of origin and cultural background. Importantly, this suggests the need to investigate how and why place of origin and ethnicity are important in shaping attitudes towards invasives—especially in touristic landscapes where tourists and their associated tourism industry are important stakeholders in invasive management (Hall et al. 2011; Anderson et al. 2015). Pissolito et al. (2020) suggest that sense of place, the emotional bond between person and place, may be important. While sense of place is generally related to visit frequency, regular visitors developing such an attachment and stronger rejection of negative environmental change in a locale, arguably many New Zealanders would have some vicarious or *in absentia* place attachment to the landscapes in the study area, and this may manifest in different attitudes towards the invasives. Halpenny (2010) refers to first time visitors’ place identity and this possibly being linked to national identity and citizenship. In this way, individuals from far away (even international tourists) may have knowledge of and attachment to particular landscapes without having physically experienced them, for example, through exposure to formal and informal touristic material.

The second aim of the study was to investigate whether knowledge of the ecological impacts of invasive plant species changed participants’ views towards the plants and their control. The study supported the view that simple environmental messaging regarding invasive species can influence attitudes toward environmental management (Bremner and Park 2007; Shackleton et al. 2007; Sharp et al. 2012; Novoa et al. 2017; Bravo-Vargas et al. 2019; Gawith 2019). The majority of participants, to some extent, changed their opinions about the invasive plant species and showed greater support for IAS control measures when the ecological impacts of the species were explained. Although, as noted above this did vary according to nationality and ethnicity.

However the caveat on the above finding is that this also varied according to species; examining the differences and similarities in responses for the two invasive plants, a considerable portion of participants did not support eradication of Russell lupins even when they were informed of the ecological impacts. This is likely related not only to Russell lupins’ attractive flowers but also to their positioning in formal and informal tourism imagery- which makes the lupins a ‘must see’ attraction for many visitors to this region of New Zealand, who have the expectation of experiencing a spectacular floral display within a scenic setting. In this way Russell lupins are actually a tourism product that visitors have paid to experience. Consequently, tourists rated Russell lupins with much higher acceptance (c.f. conifers) in the landscape, and showed greater reluctance towards the species’ control and management. This finding aligns well with previous research that suggests that support for the management of IAS which are endowed with economic value is much harder to gain (e.g. Verbrugge et al. 2013; Lindemann-Matthies 2016).

At the heart of Russell lupins’ acceptance is their attractive flowers, supporting the view that certain species traits (e.g. pretty flowers and colours) may evoke certain emotions among viewers, which may be counter to the recognition of the species as weeds, and lead to opposition to their control (Veitch and Clout 2001; Shackleton et al., 2019). This may particularly be the case for invasive plants that are also commonly cultivated (Russell lupin is a common garden plant) or look as though they could or should be in cultivation. Lindemann-Matthies (2016) concluded that even when the public has information about the invasive plant species and its impacts “they still think that the beauty of some invasive plants may in settlement areas more than outweigh the damage they may cause. In other words: beauties do not easily become beasts” (2016, p27). However, some other studies have found differently (e.g. Junge et al. 2019) i.e. that providing information on the ecological impact of invasive plants *can* lead to stronger support for control. This begs the question of what the critical factors are in transforming public acceptance of a species to public rejection. In this study, while plant traits did appear to be important in shaping individuals’ responses to the plants in question, visitors of different origin and demographic status still responded differently to each plant. This suggests that conservation managers may need to take into consideration different visitor groups when developing environmental messaging around IAS.

## Conclusion

Environmental managers need to consider the incorporation of public sentiment into the design of environmental policies and strategies (Shackleton et al. 2007; Santo et al. 2015; Shrestha et al. 2019). Simply removing certain IAS without considering public sentiment may trigger public opposition of invasion controls, thereby hampering environmental management. The study findings suggest that this may be important for management of those invasive species which form an important part of the touristic landscape and are tourism products or attractions that visitors have expectations of experiencing. But in this study, most tourists, especially international tourists, had low awareness of the ecological problems associated with IAS, suggesting a need for environmental managers to design strategies to raise tourists’ awareness, in order to improve environmentally responsible behaviours and generate support for management programmes (Shackleton et al. 2007). This may include providing sufficient scientifically credible information to tourists and building effective communication and information circulation channels (e.g. brochures, media, campaigns).

However, the findings also suggest that to treat tourists as one homogenous stakeholder group ignores the significant differences among them in terms of how they view IAS and their management. Such differences in perceptions need to be taken into account when developing the above-mentioned programmes. Further research is required to explore, for example, why Asian visitors had greater acceptance of these invasive species, whether this is culturally determined, and if so, how to develop culturally-appropriate and effective messaging regarding the management of IAS. This is particularly relevant in a destination in which visitors from Asia comprise a significant portion of total visitor numbers.

A caveat to the above is that improving knowledge about IAS alone may not translate directly into behavior change regarding IAS (Shannon et al. 2020). In the case of tourists, desirable behaviours may include, for example, being careful to avoid spreading invasive plant seeds (as observed by Ansong and Pickering 2015), taking part in or making a donation to an IAS eradication programme, or making a comment on social media to raise awareness about IAS and the need for management. Further research is required to address this knowledge-attitude-behaviour connection, and to examine what types of IAS behaviours may be considered by tourists, and whether some IAS activities may suit some groups of visitors more so than others and why.

A limitation of the study is that we made the assumption that participants were first time visitors to the study landscapes, and we did not explore the degree to which visitors had been exposed to environmental messaging about the target species. Further research could focus on what types of messages may be more effective in generating support from visitors for IAS management. It has been suggested that impact-related information may be more effective than the native/exotic distinction (van der Wal et al. 2015; Lindemann-Matthies 2016). However, conceivably, this too may vary among visitor groups—for example the exotic invasion narrative may be effective for domestic tourists, while international tourists may be more responsive to impact-related information.

Much of the information that is received by prospective visitors, however, is informal and beyond the control of invasive species managers. The role of imagery and messaging, not only from tourism organisations but from other tourists through social media, that portray certain invasive plants within the touristic landscape in a positive light, may be important in pre-socialising visitors to both expect and accept these IAS in their tourist encounters. Further research could explore how social media may be effectively deployed as a tool by conservation managers to ‘counteract’ such messages and to influence tourists’ expectations and attitudes towards IAS – contributing not only to a sense of ecological citizenship/patriotism from domestic tourists, but also to an active environmental empathy from international visitors.

## Data Availability

The datasets generated during and/or analysed during the current study are available from the corresponding author on reasonable request.
